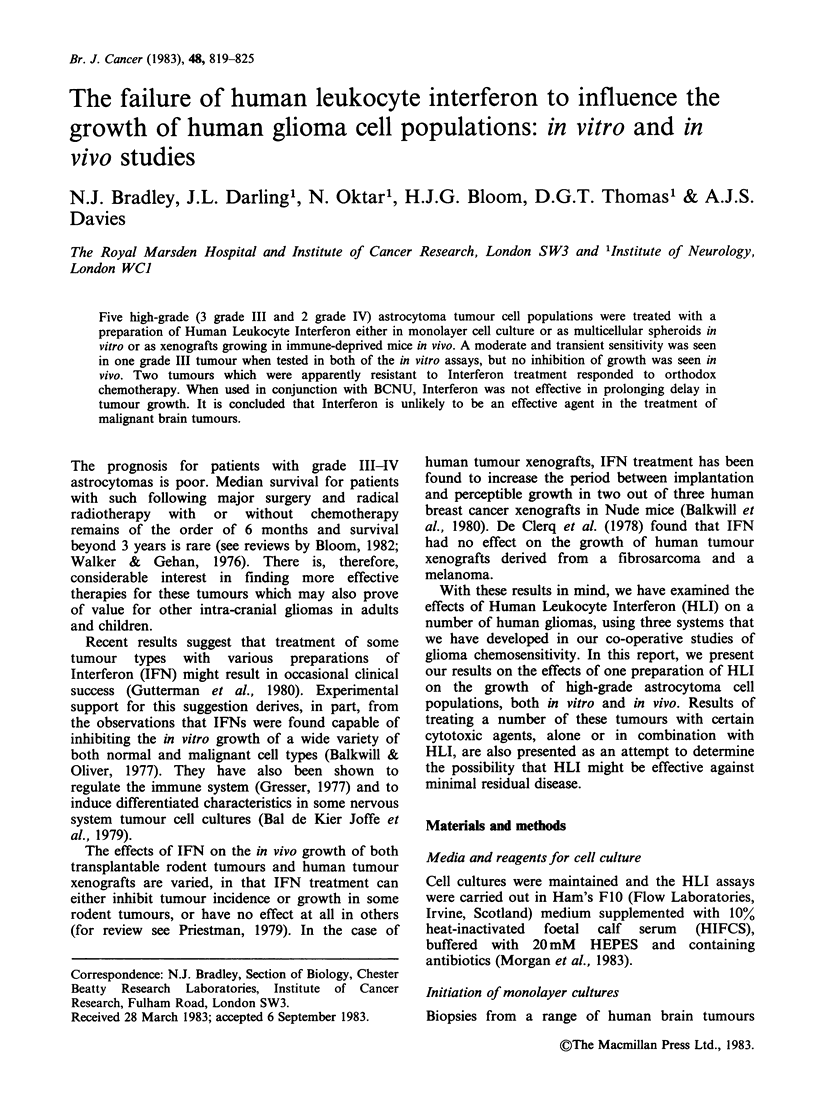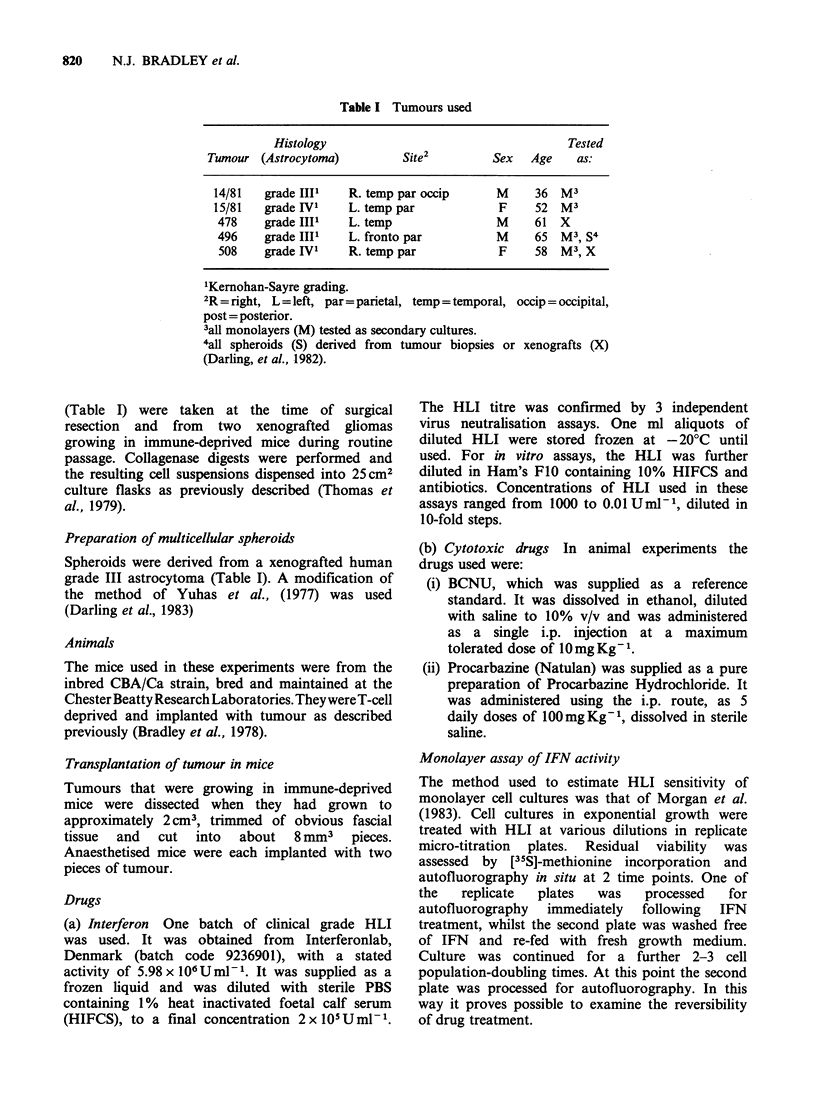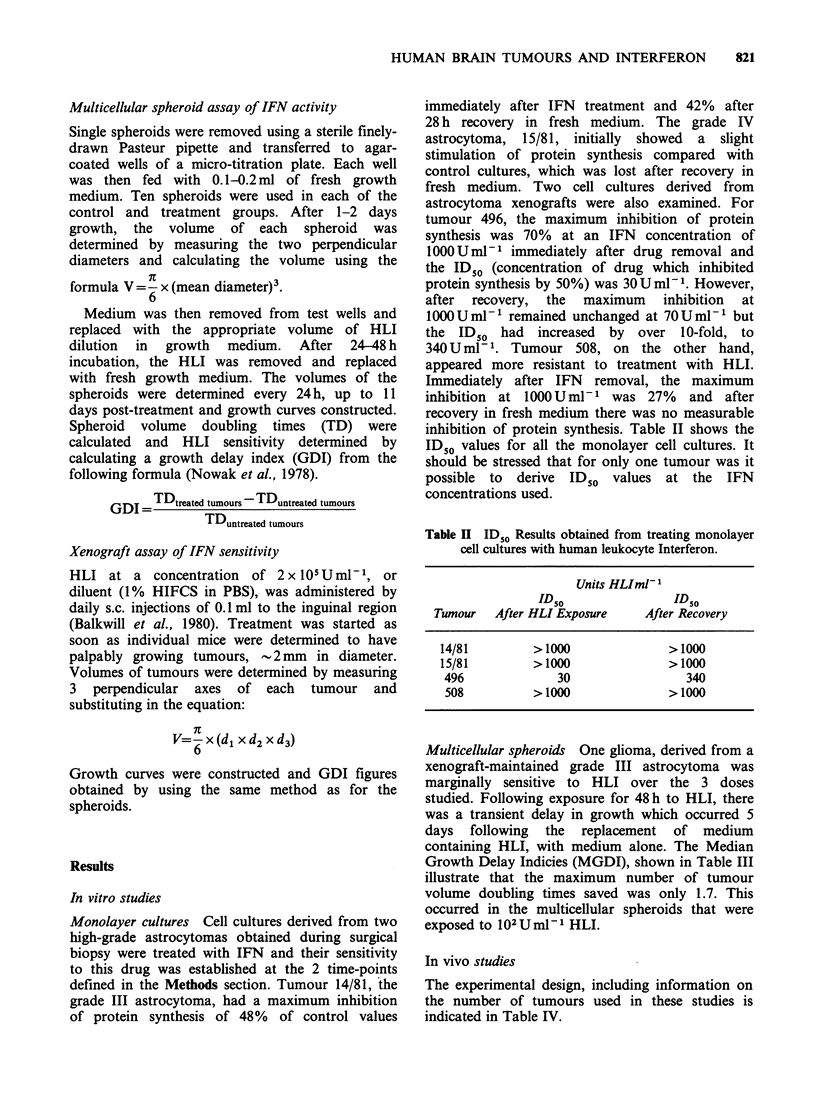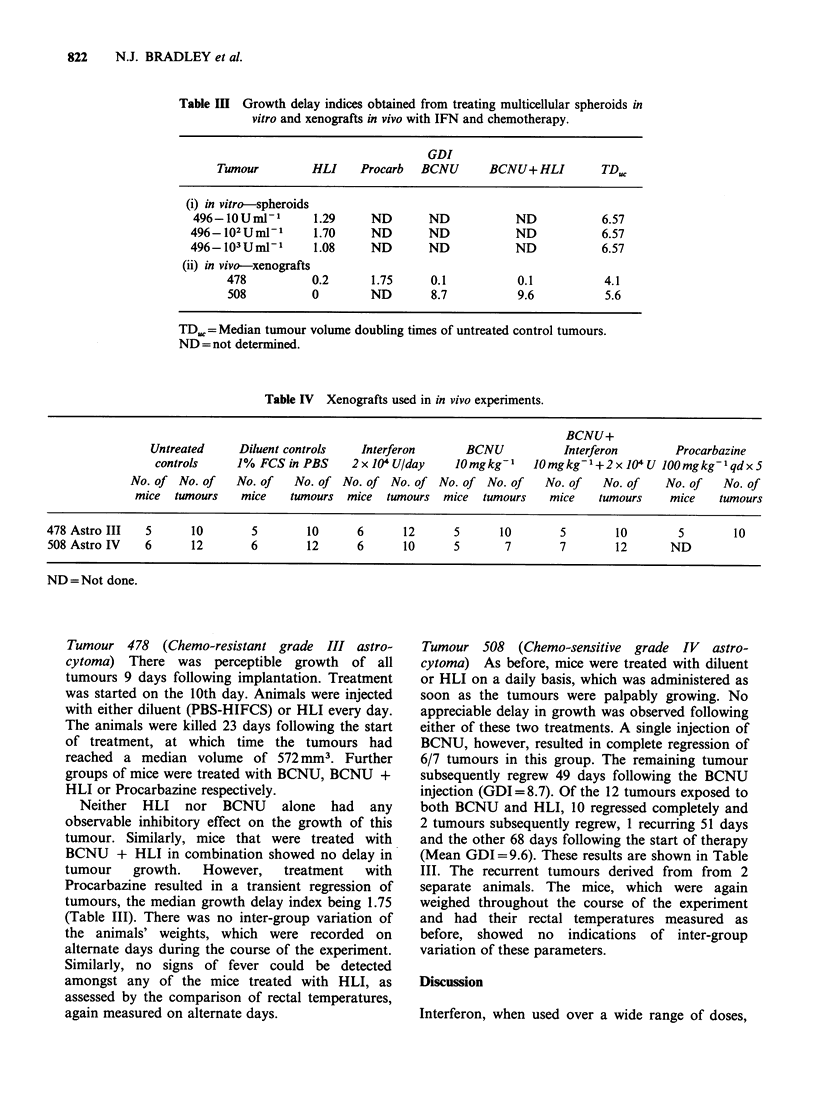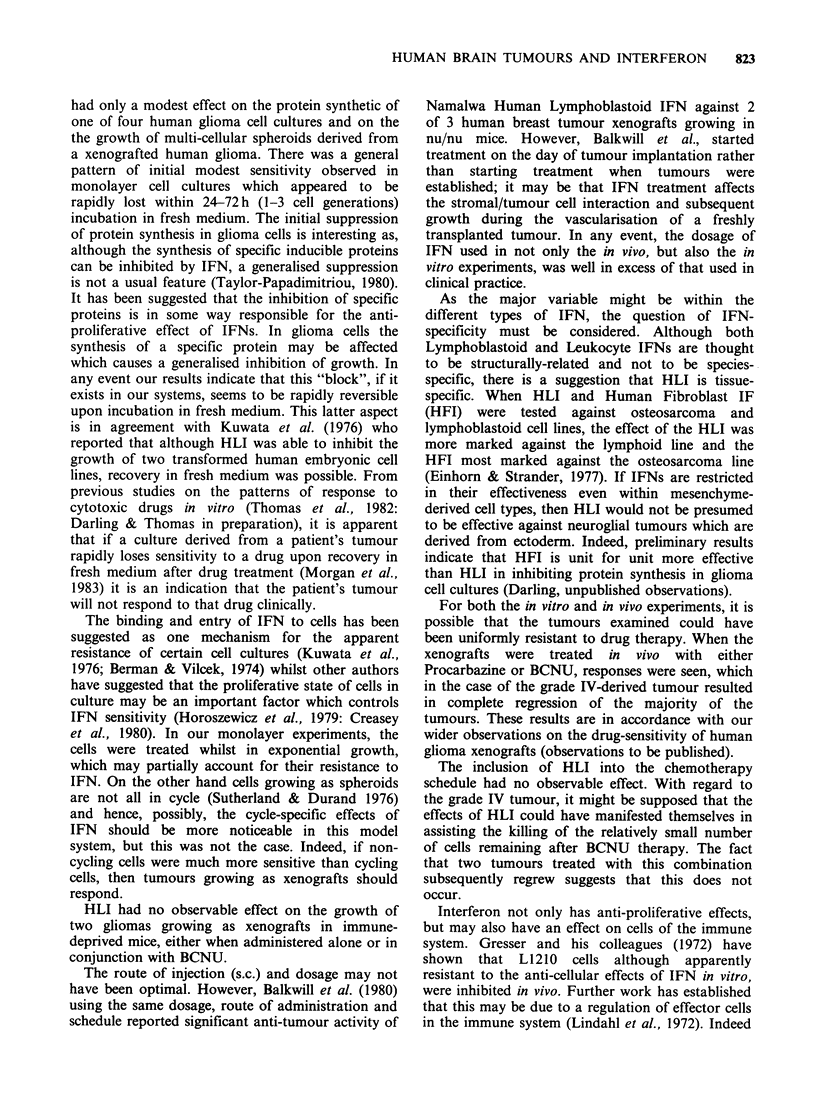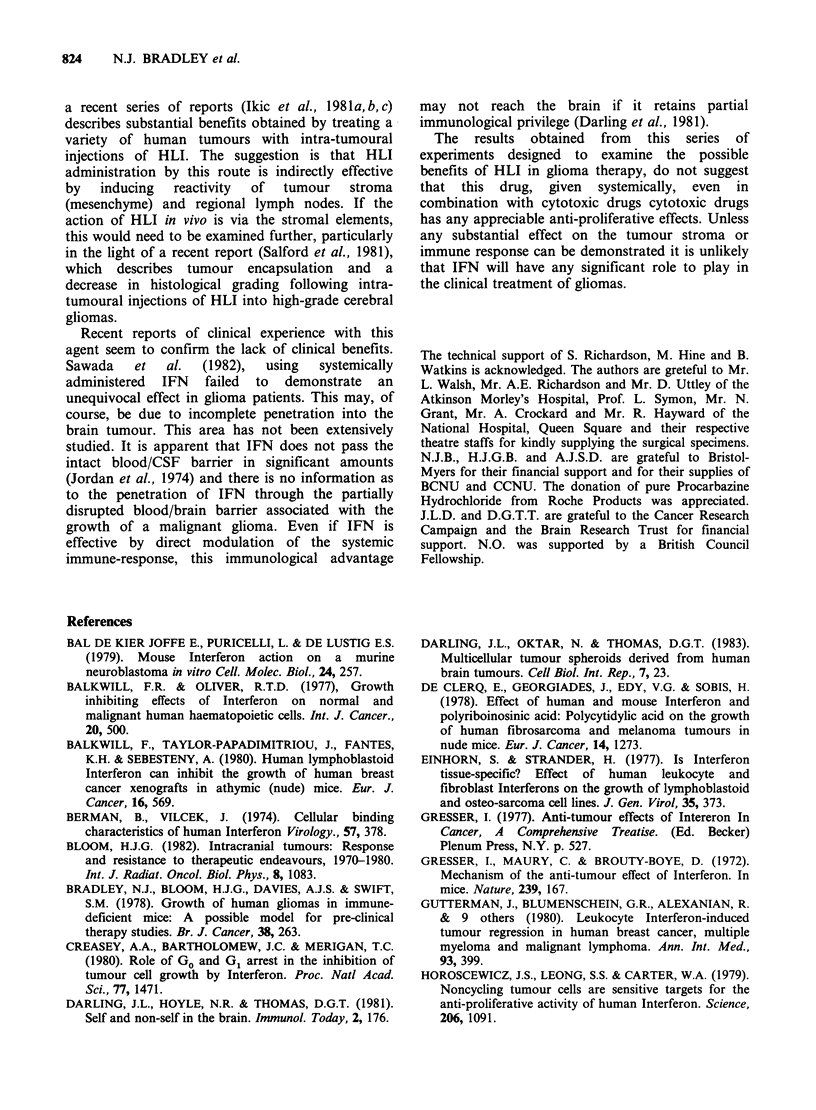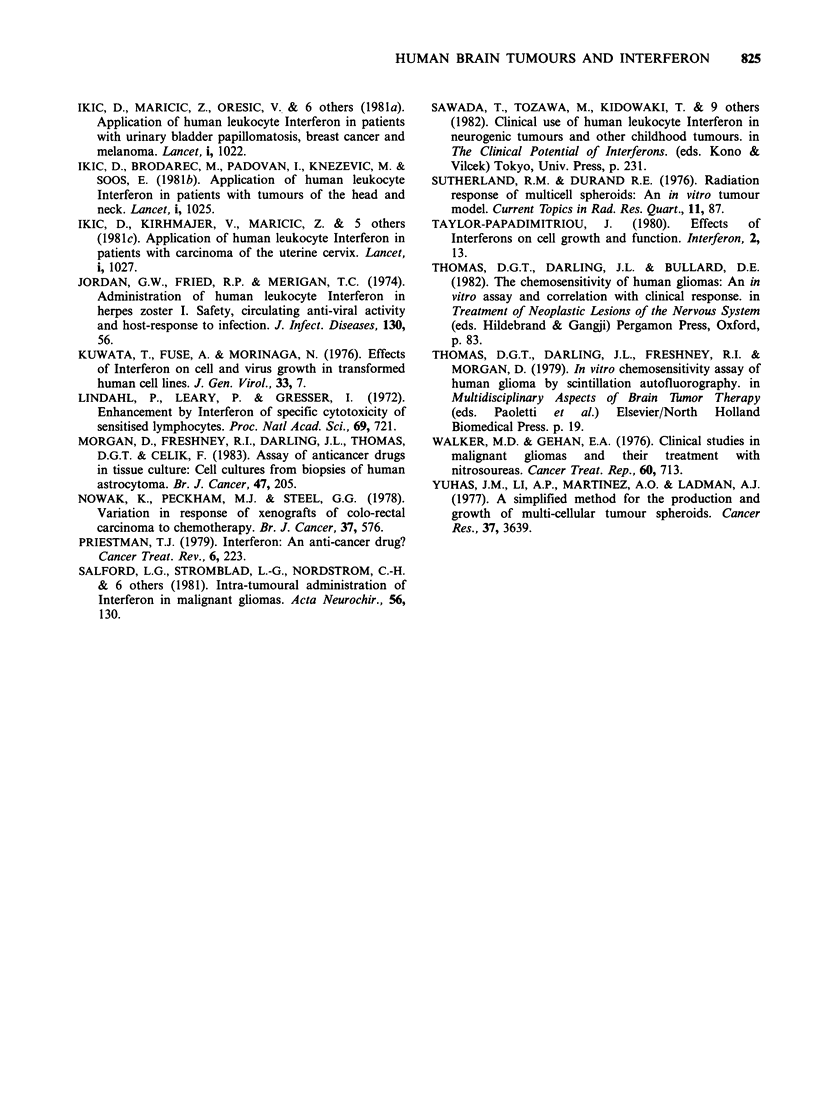# The failure of human leukocyte interferon to influence the growth of human glioma cell populations: in vitro and in vivo studies.

**DOI:** 10.1038/bjc.1983.272

**Published:** 1983-12

**Authors:** N. J. Bradley, J. L. Darling, N. Oktar, H. J. Bloom, D. G. Thomas, A. J. Davies

## Abstract

Five high-grade (3 grade III and 2 grade IV) astrocytoma tumour cell populations were treated with a preparation of Human Leukocyte Interferon either in monolayer cell culture or as multicellular spheroids in vitro or as xenografts growing in immune-deprived mice in vivo. A moderate and transient sensitivity was seen in one grade III tumour when tested in both of the in vitro assays, but no inhibition of growth was seen in vivo. Two tumours which were apparently resistant to Interferon treatment responded to orthodox chemotherapy. When used in conjunction with BCNU, Interferon was not effective in prolonging delay in tumour growth. It is concluded that Interferon is unlikely to be an effective agent in the treatment of malignant brain tumours.


*Br. J. Cancer* (1983), **48**, 819–825

### The failure of human leukocyte interferon to influence the growth of human glioma cell populations: *in vitro* and *in vivo* studies

N.J. Bradley, J.L. Darling[1], N. Oktar[1], H.J.G. Bloom, D.G.T. Thomas[1] & A.J.S. Davies

*The Royal Marsden Hospital and Institute of Cancer Research, London SW3 and [1]Institute of Neurology, London WC1*

Five high-grade (3 grade III and 2 grade IV) astrocytoma tumour cell populations were treated with a preparation of Human Leukocyte Interferon either in monolayer cell culture or as multicellular spheroids *in vitro* or as xenografts growing in immune-deprived mice *in vivo*. A moderate and transient sensitivity was seen in one grade III tumour when tested in both of the *in vitro* assays, but no inhibition of growth was seen *in vivo*. Two tumours which were apparently resistant to Interferon treatment responded to orthodox chemotherapy. When used in conjunction with BCNU, Interferon was not effective in prolonging delay in tumour growth. It is concluded that Interferon is unlikely to be an effective agent in the treatment of malignant brain tumours.

The prognosis for patients with grade III–IV astrocytomas is poor. Median survival for patients with such following major surgery and radical radiotherapy with or without chemotherapy remains of the order of 6 months and survival beyond 3 years is rare (see reviews by Bloom, 1982; Walker & Gehan, 1976). There is, therefore, considerable interest in finding more effective therapies for these tumours which may also prove of value for other intra-cranial gliomas in adults and children.

Recent results suggest that treatment of some tumour types with various preparations of Interferon (IFN) might result in occasional clinical success (Gutterman *et al.*, 1980). Experimental support for this suggestion derives, in part, from the observations that IFNs were found capable of inhibiting the *in vitro* growth of a wide variety of both normal and malignant cell types (Balkwill & Oliver, 1977). They have also been shown to regulate the immune system (Gresser, 1977) and to induce differentiated characteristics in some nervous system tumour cell cultures (Bal de Kier Joffe *et al.*, 1979).

The effects of IFN on the *in vivo* growth of both transplantable rodent tumours and human tumour xenografts are varied, in that IFN treatment can either inhibit tumour incidence or growth in some rodent tumours, or have no effect at all in others (for review see Priestman, 1979). In the case of human tumour xenografts, IFN treatment has been found to increase the period between implantation and perceptible growth in two out of three human breast cancer xenografts in Nude mice (Balkwill *et al.*, 1980). De Clerq *et al.* (1978) found that IFN had no effect on the growth of human tumour xenografts derived from a fibrosarcoma and a melanoma.

With these results in mind, we have examined the effects of Human Leukocyte Interferon (HLI) on a number of human gliomas, using three systems that we have developed in our co-operative studies of glioma chemosensitivity. In this report, we present our results on the effects of one preparation of HLI on the growth of high-grade astrocytoma cell populations, both *in vitro* and *in vivo*. Results of treating a number of these tumours with certain cytotoxic agents, alone or in combination with HLI, are also presented as an attempt to determine the possibility that HLI might be effective against minimal residual disease.

### Materials and methods

### *Media and reagents for cell culture*

Cell cultures were maintained and the HLI assays were carried out in Ham's F10 (Flow Laboratories, Irvine, Scotland) medium supplemented with 10% heat-inactivated foetal calf serum (HIFCS), buffered with 20 mM HEPES and containing antibiotics (Morgan *et al.*, 1983).

### *Initiation of monolayer cultures*

Biopsies from a range of human brain tumours

Correspondence: N.J. Bradley, Section of Biology, Chester Beatty Research Laboratories, Institute of Cancer Research, Fulham Road, London SW3.
Received 28 March 1983; accepted 6 September 1983.

©The Macmillan Press Ltd., 1983.

**Table I** Tumours used

| Tumour | Histology (Astrocytoma) | Site[2] | Sex | Age | Tested as: |
|---|---|---|---|---|---|
| 14/81 | grade III[1] | R. temp par occip | M | 36 | M[3] |
| 15/81 | grade IV[1] | L. temp par | F | 52 | M[3] |
| 478 | grade III[1] | L. temp | M | 61 | X |
| 496 | grade III[1] | L. fronto par | M | 65 | M[3], S[4] |
| 508 | grade IV[1] | R. temp par | F | 58 | M[3], X |

[1]Kernohan-Sayre grading.
[2]R = right, L = left, par = parietal, temp = temporal, occip = occipital, post = posterior.
[3]all monolayers (M) tested as secondary cultures.
[4]all spheroids (S) derived from tumour biopsies or xenografts (X) (Darling, *et al.*, 1982).

(Table I) were taken at the time of surgical resection and from two xenografted gliomas growing in immune-deprived mice during routine passage. Collagenase digests were performed and the resulting cell suspensions dispensed into 25 cm$^2$ culture flasks as previously described (Thomas *et al.*, 1979).

*Preparation of multicellular spheroids*

Spheroids were derived from a xenografted human grade III astrocytoma (Table I). A modification of the method of Yuhas *et al.*, (1977) was used (Darling *et al.*, 1983)

*Animals*

The mice used in these experiments were from the inbred CBA/Ca strain, bred and maintained at the Chester Beatty Research Laboratories. They were T-cell deprived and implanted with tumour as described previously (Bradley *et al.*, 1978).

*Transplantation of tumour in mice*

Tumours that were growing in immune-deprived mice were dissected when they had grown to approximately 2 cm$^3$, trimmed of obvious fascial tissue and cut into about 8 mm$^3$ pieces. Anaesthetised mice were each implanted with two pieces of tumour.

*Drugs*

(a) *Interferon* One batch of clinical grade HLI was used. It was obtained from Interferonlab, Denmark (batch code 9236901), with a stated activity of $5.98 \times 10^6$ U ml$^{-1}$. It was supplied as a frozen liquid and was diluted with sterile PBS containing 1% heat inactivated foetal calf serum (HIFCS), to a final concentration $2 \times 10^5$ U ml$^{-1}$. The HLI titre was confirmed by 3 independent virus neutralisation assays. One ml aliquots of diluted HLI were stored frozen at $-20$°C until used. For *in vitro* assays, the HLI was further diluted in Ham's F10 containing 10% HIFCS and antibiotics. Concentrations of HLI used in these assays ranged from 1000 to 0.01 U ml$^{-1}$, diluted in 10-fold steps.

(b) *Cytotoxic drugs* In animal experiments the drugs used were:

(i) BCNU, which was supplied as a reference standard. It was dissolved in ethanol, diluted with saline to 10% v/v and was administered as a single i.p. injection at a maximum tolerated dose of 10 mg Kg$^{-1}$.

(ii) Procarbazine (Natulan) was supplied as a pure preparation of Procarbazine Hydrochloride. It was administered using the i.p. route, as 5 daily doses of 100 mg Kg$^{-1}$, dissolved in sterile saline.

*Monolayer assay of IFN activity*

The method used to estimate HLI sensitivity of monolayer cell cultures was that of Morgan *et al.* (1983). Cell cultures in exponential growth were treated with HLI at various dilutions in replicate micro-titration plates. Residual viability was assessed by [$^{35}$S]-methionine incorporation and autofluorography *in situ* at 2 time points. One of the replicate plates was processed for autofluorography immediately following IFN treatment, whilst the second plate was washed free of IFN and re-fed with fresh growth medium. Culture was continued for a further 2–3 cell population-doubling times. At this point the second plate was processed for autofluorography. In this way it proves possible to examine the reversibility of drug treatment.

*Multicellular spheroid assay of IFN activity*

Single spheroids were removed using a sterile finely-drawn Pasteur pipette and transferred to agar-coated wells of a micro-titration plate. Each well was then fed with 0.1–0.2 ml of fresh growth medium. Ten spheroids were used in each of the control and treatment groups. After 1–2 days growth, the volume of each spheroid was determined by measuring the two perpendicular diameters and calculating the volume using the formula $V=\frac{\pi}{6}\times(\text{mean diameter})^3$.

Medium was then removed from test wells and replaced with the appropriate volume of HLI dilution in growth medium. After 24–48 h incubation, the HLI was removed and replaced with fresh growth medium. The volumes of the spheroids were determined every 24 h, up to 11 days post-treatment and growth curves constructed. Spheroid volume doubling times (TD) were calculated and HLI sensitivity determined by calculating a growth delay index (GDI) from the following formula (Nowak *et al.*, 1978).

$$\text{GDI}=\frac{\text{TD}_{\text{treated tumours}}-\text{TD}_{\text{untreated tumours}}}{\text{TD}_{\text{untreated tumours}}}$$

*Xenograft assay of IFN sensitivity*

HLI at a concentration of $2\times10^5\,\text{U ml}^{-1}$, or diluent (1% HIFCS in PBS), was administered by daily s.c. injections of 0.1 ml to the inguinal region (Balkwill *et al.*, 1980). Treatment was started as soon as individual mice were determined to have palpably growing tumours, ~2 mm in diameter. Volumes of tumours were determined by measuring 3 perpendicular axes of each tumour and substituting in the equation:

$$V=\frac{\pi}{6}\times(d_1\times d_2\times d_3)$$

Growth curves were constructed and GDI figures obtained by using the same method as for the spheroids.

### Results

*In vitro studies*

*Monolayer cultures* Cell cultures derived from two high-grade astrocytomas obtained during surgical biopsy were treated with IFN and their sensitivity to this drug was established at the 2 time-points defined in the **Methods** section. Tumour 14/81, the grade III astrocytoma, had a maximum inhibition of protein synthesis of 48% of control values immediately after IFN treatment and 42% after 28 h recovery in fresh medium. The grade IV astrocytoma, 15/81, initially showed a slight stimulation of protein synthesis compared with control cultures, which was lost after recovery in fresh medium. Two cell cultures derived from astrocytoma xenografts were also examined. For tumour 496, the maximum inhibition of protein synthesis was 70% at an IFN concentration of $1000\,\text{U ml}^{-1}$ immediately after drug removal and the $\text{ID}_{50}$ (concentration of drug which inhibited protein synthesis by 50%) was $30\,\text{U ml}^{-1}$. However, after recovery, the maximum inhibition at $1000\,\text{U ml}^{-1}$ remained unchanged at $70\,\text{U ml}^{-1}$ but the $\text{ID}_{50}$ had increased by over 10-fold, to $340\,\text{U ml}^{-1}$. Tumour 508, on the other hand, appeared more resistant to treatment with HLI. Immediately after IFN removal, the maximum inhibition at $1000\,\text{U ml}^{-1}$ was 27% and after recovery in fresh medium there was no measurable inhibition of protein synthesis. Table II shows the $\text{ID}_{50}$ values for all the monolayer cell cultures. It should be stressed that for only one tumour was it possible to derive $\text{ID}_{50}$ values at the IFN concentrations used.

**Table II** $\text{ID}_{50}$ Results obtained from treating monolayer cell cultures with human leukocyte Interferon.

| | *Units HLI ml⁻¹* | |
|---|---|---|
| *Tumour* | *$ID_{50}$ After HLI Exposure* | *$ID_{50}$ After Recovery* |
| 14/81 | >1000 | >1000 |
| 15/81 | >1000 | >1000 |
| 496 | 30 | 340 |
| 508 | >1000 | >1000 |

*Multicellular spheroids* One glioma, derived from a xenograft-maintained grade III astrocytoma was marginally sensitive to HLI over the 3 doses studied. Following exposure for 48 h to HLI, there was a transient delay in growth which occurred 5 days following the replacement of medium containing HLI, with medium alone. The Median Growth Delay Indicies (MGDI), shown in Table III illustrate that the maximum number of tumour volume doubling times saved was only 1.7. This occurred in the multicellular spheroids that were exposed to $10^2\,\text{U ml}^{-1}$ HLI.

In vivo *studies*

The experimental design, including information on the number of tumours used in these studies is indicated in Table IV.

**Table III** Growth delay indices obtained from treating multicellular spheroids *in vitro* and xenografts *in vivo* with IFN and chemotherapy.

| *Tumour* | *HLI* | *Procarb* | *GDI BCNU* | *BCNU+HLI* | $TD_{uc}$ |
|---|---|---|---|---|---|
| (i) *in vitro*—spheroids | | | | | |
| $496-10\,U\,ml^{-1}$ | 1.29 | ND | ND | ND | 6.57 |
| $496-10^2\,U\,ml^{-1}$ | 1.70 | ND | ND | ND | 6.57 |
| $496-10^3\,U\,ml^{-1}$ | 1.08 | ND | ND | ND | 6.57 |
| (ii) *in vivo*—xenografts | | | | | |
| 478 | 0.2 | 1.75 | 0.1 | 0.1 | 4.1 |
| 508 | 0 | ND | 8.7 | 9.6 | 5.6 |

$TD_{uc}$ = Median tumour volume doubling times of untreated control tumours.
ND = not determined.

**Table IV** Xenografts used in *in vivo* experiments.

| | *Untreated controls* | | *Diluent controls 1% FCS in PBS* | | *Interferon 2×10⁴ U/day* | | *BCNU 10 mg kg⁻¹* | | *BCNU+ Interferon 10 mg kg⁻¹+2×10⁴ U* | | *Procarbazine 100 mg kg⁻¹ qd×5* | |
|---|---|---|---|---|---|---|---|---|---|---|---|---|
| | *No. of mice* | *No. of tumours* | *No. of mice* | *No. of tumours* | *No. of mice* | *No. of tumours* | *No. of mice* | *No. of tumours* | *No. of mice* | *No. of tumours* | *No. of mice* | *No. of tumours* |
| 478 Astro III | 5 | 10 | 5 | 10 | 6 | 12 | 5 | 10 | 5 | 10 | 5 | 10 |
| 508 Astro IV | 6 | 12 | 6 | 12 | 6 | 10 | 5 | 7 | 7 | 12 | ND | |

ND = Not done.

*Tumour 478 (Chemo-resistant grade III astrocytoma)* There was perceptible growth of all tumours 9 days following implantation. Treatment was started on the 10th day. Animals were injected with either diluent (PBS-HIFCS) or HLI every day. The animals were killed 23 days following the start of treatment, at which time the tumours had reached a median volume of $572\,mm^3$. Further groups of mice were treated with BCNU, BCNU + HLI or Procarbazine respectively.

Neither HLI nor BCNU alone had any observable inhibitory effect on the growth of this tumour. Similarly, mice that were treated with BCNU + HLI in combination showed no delay in tumour growth. However, treatment with Procarbazine resulted in a transient regression of tumours, the median growth delay index being 1.75 (Table III). There was no inter-group variation of the animals' weights, which were recorded on alternate days during the course of the experiment. Similarly, no signs of fever could be detected amongst any of the mice treated with HLI, as assessed by the comparison of rectal temperatures, again measured on alternate days.

*Tumour 508 (Chemo-sensitive grade IV astrocytoma)* As before, mice were treated with diluent or HLI on a daily basis, which was administered as soon as the tumours were palpably growing. No appreciable delay in growth was observed following either of these two treatments. A single injection of BCNU, however, resulted in complete regression of 6/7 tumours in this group. The remaining tumour subsequently regrew 49 days following the BCNU injection (GDI = 8.7). Of the 12 tumours exposed to both BCNU and HLI, 10 regressed completely and 2 tumours subsequently regrew, 1 recurring 51 days and the other 68 days following the start of therapy (Mean GDI = 9.6). These results are shown in Table III. The recurrent tumours derived from from 2 separate animals. The mice, which were again weighed throughout the course of the experiment and had their rectal temperatures measured as before, showed no indications of inter-group variation of these parameters.

### Discussion

Interferon, when used over a wide range of doses, had only a modest effect on the protein synthetic of one of four human glioma cell cultures and on the the growth of multi-cellular spheroids derived from a xenografted human glioma. There was a general pattern of initial modest sensitivity observed in monolayer cell cultures which appeared to be rapidly lost within 24–72 h (1–3 cell generations) incubation in fresh medium. The initial suppression of protein synthesis in glioma cells is interesting as, although the synthesis of specific inducible proteins can be inhibited by IFN, a generalised suppression is not a usual feature (Taylor-Papadimitriou, 1980). It has been suggested that the inhibition of specific proteins is in some way responsible for the anti-proliferative effect of IFNs. In glioma cells the synthesis of a specific protein may be affected which causes a generalised inhibition of growth. In any event our results indicate that this "block", if it exists in our systems, seems to be rapidly reversible upon incubation in fresh medium. This latter aspect is in agreement with Kuwata *et al.* (1976) who reported that although HLI was able to inhibit the growth of two transformed human embryonic cell lines, recovery in fresh medium was possible. From previous studies on the patterns of response to cytotoxic drugs *in vitro* (Thomas *et al.*, 1982: Darling & Thomas in preparation), it is apparent that if a culture derived from a patient's tumour rapidly loses sensitivity to a drug upon recovery in fresh medium after drug treatment (Morgan *et al.*, 1983) it is an indication that the patient's tumour will not respond to that drug clinically.

The binding and entry of IFN to cells has been suggested as one mechanism for the apparent resistance of certain cell cultures (Kuwata *et al.*, 1976; Berman & Vilcek, 1974) whilst other authors have suggested that the proliferative state of cells in culture may be an important factor which controls IFN sensitivity (Horoszewicz *et al.*, 1979: Creasey *et al.*, 1980). In our monolayer experiments, the cells were treated whilst in exponential growth, which may partially account for their resistance to IFN. On the other hand cells growing as spheroids are not all in cycle (Sutherland & Durand 1976) and hence, possibly, the cycle-specific effects of IFN should be more noticeable in this model system, but this was not the case. Indeed, if non-cycling cells were much more sensitive than cycling cells, then tumours growing as xenografts should respond.

HLI had no observable effect on the growth of two gliomas growing as xenografts in immune-deprived mice, either when administered alone or in conjunction with BCNU.

The route of injection (s.c.) and dosage may not have been optimal. However, Balkwill *et al.* (1980) using the same dosage, route of administration and schedule reported significant anti-tumour activity of Namalwa Human Lymphoblastoid IFN against 2 of 3 human breast tumour xenografts growing in nu/nu mice. However, Balkwill *et al.*, started treatment on the day of tumour implantation rather than starting treatment when tumours were established; it may be that IFN treatment affects the stromal/tumour cell interaction and subsequent growth during the vascularisation of a freshly transplanted tumour. In any event, the dosage of IFN used in not only the *in vivo,* but also the *in vitro* experiments, was well in excess of that used in clinical practice.

As the major variable might be within the different types of IFN, the question of IFN-specificity must be considered. Although both Lymphoblastoid and Leukocyte IFNs are thought to be structurally-related and not to be species-specific, there is a suggestion that HLI is tissue-specific. When HLI and Human Fibroblast IF (HFI) were tested against osteosarcoma and lymphoblastoid cell lines, the effect of the HLI was more marked against the lymphoid line and the HFI most marked against the osteosarcoma line (Einhorn & Strander, 1977). If IFNs are restricted in their effectiveness even within mesenchyme-derived cell types, then HLI would not be presumed to be effective against neuroglial tumours which are derived from ectoderm. Indeed, preliminary results indicate that HFI is unit for unit more effective than HLI in inhibiting protein synthesis in glioma cell cultures (Darling, unpublished observations).

For both the *in vitro* and *in vivo* experiments, it is possible that the tumours examined could have been uniformly resistant to drug therapy. When the xenografts were treated *in vivo* with either Procarbazine or BCNU, responses were seen, which in the case of the grade IV-derived tumour resulted in complete regression of the majority of the tumours. These results are in accordance with our wider observations on the drug-sensitivity of human glioma xenografts (observations to be published).

The inclusion of HLI into the chemotherapy schedule had no observable effect. With regard to the grade IV tumour, it might be supposed that the effects of HLI could have manifested themselves in assisting the killing of the relatively small number of cells remaining after BCNU therapy. The fact that two tumours treated with this combination subsequently regrew suggests that this does not occur.

Interferon not only has anti-proliferative effects, but may also have an effect on cells of the immune system. Gresser and his colleagues (1972) have shown that L1210 cells although apparently resistant to the anti-cellular effects of IFN *in vitro,* were inhibited *in vivo*. Further work has established that this may be due to a regulation of effector cells in the immune system (Lindahl *et al.*, 1972). Indeed a recent series of reports (Ikic *et al.*, 1981*a,b,c*) describes substantial benefits obtained by treating a variety of human tumours with intra-tumoural injections of HLI. The suggestion is that HLI administration by this route is indirectly effective by inducing reactivity of tumour stroma (mesenchyme) and regional lymph nodes. If the action of HLI *in vivo* is via the stromal elements, this would need to be examined further, particularly in the light of a recent report (Salford *et al.*, 1981), which describes tumour encapsulation and a decrease in histological grading following intra-tumoural injections of HLI into high-grade cerebral gliomas.

Recent reports of clinical experience with this agent seem to confirm the lack of clinical benefits. Sawada *et al.* (1982), using systemically administered IFN failed to demonstrate an unequivocal effect in glioma patients. This may, of course, be due to incomplete penetration into the brain tumour. This area has not been extensively studied. It is apparent that IFN does not pass the intact blood/CSF barrier in significant amounts (Jordan *et al.*, 1974) and there is no information as to the penetration of IFN through the partially disrupted blood/brain barrier associated with the growth of a malignant glioma. Even if IFN is effective by direct modulation of the systemic immune-response, this immunological advantage may not reach the brain if it retains partial immunological privilege (Darling *et al.*, 1981).

The results obtained from this series of experiments designed to examine the possible benefits of HLI in glioma therapy, do not suggest that this drug, given systemically, even in combination with cytotoxic drugs cytotoxic drugs has any appreciable anti-proliferative effects. Unless any substantial effect on the tumour stroma or immune response can be demonstrated it is unlikely that IFN will have any significant role to play in the clinical treatment of gliomas.

The technical support of S. Richardson, M. Hine and B. Watkins is acknowledged. The authors are greteful to Mr. L. Walsh, Mr. A.E. Richardson and Mr. D. Uttley of the Atkinson Morley's Hospital, Prof. L. Symon, Mr. N. Grant, Mr. A. Crockard and Mr. R. Hayward of the National Hospital, Queen Square and their respective theatre staffs for kindly supplying the surgical specimens. N.J.B., H.J.G.B. and A.J.S.D. are grateful to Bristol-Myers for their financial support and for their supplies of BCNU and CCNU. The donation of pure Procarbazine Hydrochloride from Roche Products was appreciated. J.L.D. and D.G.T.T. are grateful to the Cancer Research Campaign and the Brain Research Trust for financial support. N.O. was supported by a British Council Fellowship.

### References

BAL DE KIER JOFFE E., PURICELLI, L. & DE LUSTIG E.S. (1979). Mouse Interferon action on a murine neuroblastoma *in vitro Cell. Molec. Biol.*, **24**, 257.

BALKWILL, F.R. & OLIVER, R.T.D. (1977), Growth inhibiting effects of Interferon on normal and malignant human haematopoietic cells. *Int. J. Cancer.*, **20**, 500.

BALKWILL, F., TAYLOR-PAPADIMITRIOU, J., FANTES, K.H. & SEBESTENY, A. (1980). Human lymphoblastoid Interferon can inhibit the growth of human breast cancer xenografts in athymic (nude) mice. *Eur. J. Cancer*, **16**, 569.

BERMAN, B., VILCEK, J. (1974). Cellular binding characteristics of human Interferon *Virology.*, **57**, 378.

BLOOM, H.J.G. (1982). Intracranial tumours: Response and resistance to therapeutic endeavours, 1970–1980. *Int. J. Radiat. Oncol. Biol. Phys.*, **8**, 1083.

BRADLEY, N.J., BLOOM, H.J.G., DAVIES, A.J.S. & SWIFT, S.M. (1978). Growth of human gliomas in immune-deficient mice: A possible model for pre-clinical therapy studies. *Br. J. Cancer*, **38**, 263.

CREASEY, A.A., BARTHOLOMEW, J.C. & MERIGAN, T.C. (1980). Role of $G_0$ and $G_1$ arrest in the inhibition of tumour cell growth by Interferon. *Proc. Natl Acad. Sci.*, **77**, 1471.

DARLING, J.L., HOYLE, N.R. & THOMAS, D.G.T. (1981). Self and non-self in the brain. *Immunol. Today*, **2**, 176.

DARLING, J.L., OKTAR, N. & THOMAS, D.G.T. (1983). Multicellular tumour spheroids derived from human brain tumours. *Cell Biol. Int. Rep.*, **7**, 23.

DE CLERQ, E., GEORGIADES, J., EDY, V.G. & SOBIS, H. (1978). Effect of human and mouse Interferon and polyriboinosinic acid: Polycytidylic acid on the growth of human fibrosarcoma and melanoma tumours in nude mice. *Eur. J. Cancer*, **14**, 1273.

EINHORN, S. & STRANDER, H. (1977). Is Interferon tissue-specific? Effect of human leukocyte and fibroblast Interferons on the growth of lymphoblastoid and osteo-sarcoma cell lines. *J. Gen. Virol*, **35**, 373.

GRESSER, I. (1977). Anti-tumour effects of Intereron In *Cancer, A Comprehensive Treatise*. (Ed. Becker) Plenum Press, N.Y. p. 527.

GRESSER, I., MAURY, C. & BROUTY-BOYE, D. (1972). Mechanism of the anti-tumour effect of Interferon. In mice. *Nature*, **239**, 167.

GUTTERMAN, J., BLUMENSCHEIN, G.R., ALEXANIAN, R. & 9 others (1980). Leukocyte Interferon-induced tumour regression in human breast cancer, multiple myeloma and malignant lymphoma. *Ann. Int. Med.*, **93**, 399.

HOROSCEWICZ, J.S., LEONG, S.S. & CARTER, W.A. (1979). Noncycling tumour cells are sensitive targets for the anti-proliferative activity of human Interferon. *Science*, **206**, 1091.

IKIC, D., MARICIC, Z., ORESIC, V. & 6 others (1981*a*). Application of human leukocyte Interferon in patients with urinary bladder papillomatosis, breast cancer and melanoma. *Lancet*, **i**, 1022.

IKIC, D., BRODAREC, M., PADOVAN, I., KNEZEVIC, M. & SOOS, E. (1981*b*). Application of human leukocyte Interferon in patients with tumours of the head and neck. *Lancet*, **i**, 1025.

IKIC, D., KIRHMAJER, V., MARICIC, Z. & 5 others (1981*c*). Application of human leukocyte Interferon in patients with carcinoma of the uterine cervix. *Lancet*, **i**, 1027.

JORDAN, G.W., FRIED, R.P. & MERIGAN, T.C. (1974). Administration of human leukocyte Interferon in herpes zoster I. Safety, circulating anti-viral activity and host-response to infection. *J. Infect. Diseases*, **130**, 56.

KUWATA, T., FUSE, A. & MORINAGA, N. (1976). Effects of Interferon on cell and virus growth in transformed human cell lines. *J. Gen. Virol.*, **33**, 7.

LINDAHL, P., LEARY, P. & GRESSER, I. (1972). Enhancement by Interferon of specific cytotoxicity of sensitised lymphocytes. *Proc. Natl Acad. Sci.*, **69**, 721.

MORGAN, D., FRESHNEY, R.I., DARLING, J.L., THOMAS, D.G.T. & CELIK, F. (1983). Assay of anticancer drugs in tissue culture: Cell cultures from biopsies of human astrocytoma. *Br. J. Cancer*, **47**, 205.

NOWAK, K., PECKHAM, M.J. & STEEL, G.G. (1978). Variation in response of xenografts of colo-rectal carcinoma to chemotherapy. *Br. J. Cancer*, **37**, 576.

PRIESTMAN, T.J. (1979). Interferon: An anti-cancer drug? *Cancer Treat. Rev.*, **6**, 223.

SALFORD, L.G., STROMBLAD, L.-G., NORDSTROM, C.-H. & 6 others (1981). Intra-tumoural administration of Interferon in malignant gliomas. *Acta Neurochir.*, **56**, 130.

SAWADA, T., TOZAWA, M., KIDOWAKI, T. & 9 others (1982). Clinical use of human leukocyte Interferon in neurogenic tumours and other childhood tumours. in *The Clinical Potential of Interferons*. (eds. Kono & Vilcek) Tokyo, Univ. Press, p. 231.

SUTHERLAND, R.M. & DURAND R.E. (1976). Radiation response of multicell spheroids: An *in vitro* tumour model. *Current Topics in Rad. Res. Quart.*, **11**, 87.

TAYLOR-PAPADIMITRIOU, J. (1980). Effects of Interferons on cell growth and function. *Interferon*, **2**, 13.

THOMAS, D.G.T., DARLING, J.L. & BULLARD, D.E. (1982). The chemosensitivity of human gliomas: An *in vitro* assay and correlation with clinical response. in *Treatment of Neoplastic Lesions of the Nervous System* (eds. Hildebrand & Gangji) Pergamon Press, Oxford, p. 83.

THOMAS, D.G.T., DARLING, J.L., FRESHNEY, R.I. & MORGAN, D. (1979). *In vitro* chemosensitivity assay of human glioma by scintillation autofluorography. in *Multidisciplinary Aspects of Brain Tumor Therapy* (eds. Paoletti *et al.*) Elsevier/North Holland Biomedical Press. p. 19.

WALKER, M.D. & GEHAN, E.A. (1976). Clinical studies in malignant gliomas and their treatment with nitrosoureas. *Cancer Treat. Rep.*, **60**, 713.

YUHAS, J.M., LI, A.P., MARTINEZ, A.O. & LADMAN, A.J. (1977). A simplified method for the production and growth of multi-cellular tumour spheroids. *Cancer Res.*, **37**, 3639.